# Cytomegalovirus Management in Solid Organ Transplant Recipients: A Pre-COVID-19 Survey From the Working Group of the European Society for Organ Transplantation

**DOI:** 10.3389/ti.2022.10332

**Published:** 2022-06-22

**Authors:** Paolo Antonio Grossi, Nassim Kamar, Faouzi Saliba, Fausto Baldanti, Jose M. Aguado, Jens Gottlieb, Bernhard Banas, Luciano Potena

**Affiliations:** ^1^ Department of Medicine and Surgery, University of Insubria, ASST-Sette Laghi, Varese, Italy; ^2^ Department of Nephrology and Organ Transplantation, CHU Rangueil, Université Paul Sabatier, Toulouse, France; ^3^ AP-HP Hôpital Paul Brousse, Center Hépato-Biliaire, Université Paris-Saclay, INSERM Unit N°1193, Villejuif, France; ^4^ Department of Clinical, Surgical, Diagnostic and Pediatric Sciences, University of Pavia, Pavia, Italy; ^5^ Molecular Virology Unit, Fondazione IRCCS Policlinico San Matteo, Pavia, Italy; ^6^ Unit of Infectious Diseases, Hospital Universitario “12 de Octubre”, School of Medicine, Universidad Complutense, Madrid, Spain; ^7^ Department of Respiratory Medicine, Hannover Medical School, Hannover, Germany; ^8^ Department of Nephrology, University Hospital Regensburg, Regensburg, Germany; ^9^ Heart Failure and Transplant Unit, IRCCS Azienda Ospedaliero-Universitaria di Bologna, Bologna, Italy

**Keywords:** survey, organ transplantation, infection cytomegalovirus, prophylaxis, pre-emptive therapy, cellular immunity, ESOT

## Abstract

Infections are leading causes of morbidity/mortality following solid organ transplantation (SOT) and cytomegalovirus (CMV) is among the most frequent pathogens, causing a considerable threat to SOT recipients. A survey was conducted 19 July–31 October 2019 to capture clinical practices about CMV in SOT recipients (e.g., how practices aligned with guidelines, how adequately treatments met patients’ needs, and respondents’ expectations for future developments). Transplant professionals completed a ∼30-minute online questionnaire: 224 responses were included, representing 160 hospitals and 197 SOT programs (41 countries; 167[83%] European programs). Findings revealed a heterogenous approach to CMV diagnosis and management and, sometimes, significant divergence from international guidelines. Valganciclovir prophylaxis (of variable duration) was administered by 201/224 (90%) respondents in D+/R− SOT and by 40% in R+ cases, with pre-emptive strategies generally reserved for R+ cases: DNA thresholds to initiate treatment ranged across 10–10,000 copies/ml. Ganciclovir-resistant CMV strains were still perceived as major challenges, and tailored treatment was one of the most important unmet needs for CMV management. These findings may help to design studies to evaluate safety and efficacy of new strategies to prevent CMV disease in SOT recipients, and target specific educational activities to harmonize CMV management in this challenging population.

## GRAPHICAL ABSTRACT

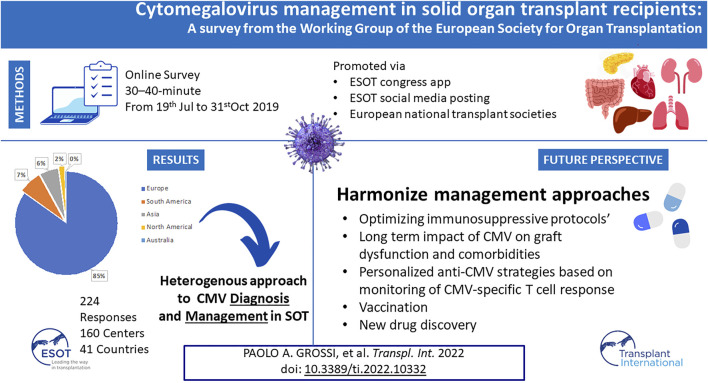

## Introduction

Cytomegalovirus (CMV) is one of the most important opportunistic viral pathogens in the solid organ transplant (SOT) setting (1–3); CMV infection and disease (defined as evidence of infection with attributable symptoms (4)) can cause adverse outcomes for allograft and recipient survival, increase the cost of transplantation, and negatively impact health-related quality of life (1–3).

Pre-transplant CMV immunoglobulin (Ig)G serological testing is generally undertaken in both donor and recipient to establish CMV disease risk and guide infection prevention strategies (5–9). CMV‐seronegative recipients who receive organs from CMV‐seropositive donors (D+/R−) are at highest risk of disease since they lack the ability to mount an effective and timely immune response, because of pharmacological immunosuppression post-transplantation (10). CMV-seropositive recipients may also experience CMV reactivation and/or reinfection in up to 20% of cases, representing an “intermediate risk” subgroup (11, 12).

Two approaches that reduce risk of CMV infection and disease following SOT are universal prophylactic therapy for all “at-risk” patients (excluding D−/R−), and pre-emptive antiviral treatment (PET) for those with evidence of infection but no overt disease (8, 9, 13, 14). Despite universal prophylaxis, CMV disease can arise following discontinuation of antiviral prophylaxis, or because of resistance to antiviral treatment, with breakthrough CMV infections occurring in patients on antiviral prophylaxis (1).

The CMV-DNA polymerase inhibitors ganciclovir and valganciclovir are first-line agents for CMV prevention and treatment; foscarnet and cidofovir are reserved for refractory/resistant infections. Although these therapies are generally efficacious in SOT recipients, their clinical value is limited by their toxicity profiles: adverse events observed include myelosuppression (ganciclovir and valganciclovir), nephrotoxicity (foscarnet and cidofovir), and electrolyte imbalances (foscarnet) (15).

Regular post-transplant monitoring of viral replication helps to predict CMV disease risk and guide decisions relating to treatment duration and efficacy (8, 9, 16–18). Monitoring was traditionally undertaken with the pp65 antigenemia assay and qualitative polymerase chain reaction (PCR) (7, 19), but there has been a shift toward molecular methods such as quantitative nucleic acid testing. In addition, monitoring CMV-specific T cell immunity post-transplantation is an emerging tool for predicting and controlling CMV infection, and for guiding tailored prevention strategies (8, 9, 16, 20). CMV has a broad impact across the immune system (20–22), with the T cell-mediated adaptive immune response being predominant in conferring protection against CMV-related disease.

Despite these apparently successful approaches for managing risk of CMV disease in SOT recipients, a retrospective analysis of French data from 2007 to 2011 involving 20,473 SOT recipients demonstrated that ∼12% developed CMV disease within 24 months post transplantation (1). CMV disease was significantly associated with increased risk of allograft rejection and mortality (1). These findings demonstrate the continuing burden of CMV disease in SOT recipients and indicate the ongoing need to improve clinical outcomes for these people.

To better understand current international practices within transplant professionals, the European Society for Organ Transplantation (ESOT) conducted a survey that aimed to characterize strategies used to prevent, diagnose, and treat CMV infection in SOT recipients. The survey also sought to analyze variations in clinical practice by organ type and donor/recipient match, and to investigate drivers for variations in the use of immunosuppressive therapy regimens. Monitoring CMV-specific T cell immunity was also investigated. It was anticipated that the survey findings might influence the design of prospective multicenter studies, and identify educational needs of the transplant professional community, to help improve CMV management in SOT recipients.

## Materials and Methods

This was a questionnaire-based, cross-sectional online study, devised by ESOT and undertaken among the ESOT transplant professional’s community. A Working Group was established in May 2019 to develop, refine, conduct, interpret, and publish this survey: group members were selected by the ESOT executive council, based on specific expertise in the management of CMV in SOT rather than by geographic location or nationality. A key objective was to involve experts from the field of infectious diseases and those with organ-specific expertise, including multiorgan transplantation.

The Working Group developed the questionnaire via several rounds of in-person and virtual discussion/revision, and the content was ratified in a virtual meeting in June 2019. The final survey consisted of 57 questions, including structured (multiple-choice) and open-ended questions (Supplementary Table S1), which took ∼30–40 min to complete. The survey was hosted on cloud-based software (SurveyMonkey®, San Mateo, CA, United States) between 19 July and 31 October 2019.

The survey was promoted via a targeted online newsletter to all persons in the ESOT contact database via the congress app during the ESOT 2019 congress in Copenhagen, Denmark (September 2019), and via ESOT social media postings. European scientific organizations with an infectious disease focus [e.g., ESCMID (European Society of Clinical Microbiology and Infectious Diseases)] and European national transplant societies were also asked to promote the survey among their members.

The survey could be completed by any respondent, provided they had direct experience with managing CMV infection. Because the focus of the survey was the practice, knowledge, and opinions of the single transplant professional, responses from multiple personnel from the same institute were permitted. Questions 1–4, which focused on the respondent’s specialty area and length of active clinical practice, were included to eliminate practitioners who did not declare appropriate experience.

Participants were provided with information outlining the survey objectives prior to their involvement. Those who answered all questions were offered 1 year’s free access to the ESOT e-learning platform, *Transplant Live*, *via* a promotional code. No personal details were requested of participants, to maintain confidentiality.

Request for authorization by the ethics committee at each center was deemed unnecessary: the survey was only intended to collect the personal perceptions/opinions of transplant professionals and neither directly involved patients nor sought patient-specific data.

### Statistical Analyses

All fully completed surveys were included in the analysis. Summary statistics were generated from SurveyMonkey®. Raw data were downloaded onto an Excel® (Microsoft Office, Redmond, WA, United States) spreadsheet, for subsequent analysis. Continuous variables are reported as mean ± SD or median; categorical variables are reported as %. Most of the questions returned categorical answers, therefore for the scope of this manuscript, between-group differences were analyzed using the χ^2^ test (PRISM 7; GraphPad Software Inc., San Diego, CA, United States). *p*-values <0.05 were considered statistically significant.

For several questions, respondents were asked to grade their opinions regarding specific statements by using a 1–7 ranking, where 1 indicated maximum disagreement and 7 indicated highest agreement. Responses were analyzed by calculating the weighted average for each item: if the weighted average was >5, we assumed consensus for that statement; if it was <3, we assumed consensus against; scores between 3 and 5 were interpreted as no consensus.

## Results

### Respondent Demographics

**FIGURE 1:**
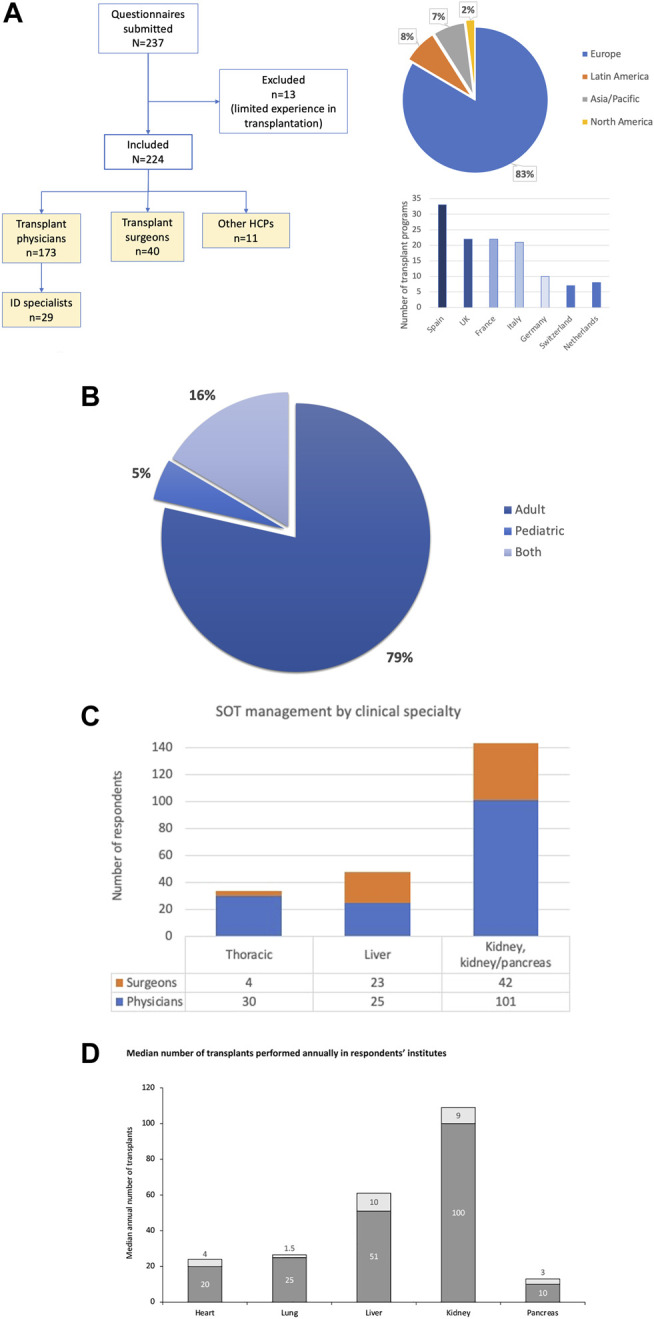
Disposition of study respondents according to medical specialty, and geographic distribution of respective transplant programs **(A)**; transplantation practices within institutions **(B**,**C)**; and median numbers of annual transplantations performed in respondents’ institutions **(D)**. The 11 “other” HCPs included immunologists, hematologists, intensivists, pharmacologists and nurse practitioners.

Disposition of study respondents is shown in Figure 1A. Of the 160 institutions represented, 128 (80%) were European, and most were responsible for multiple transplant programs. Survey responses represented 41 countries in Europe, South America, Asia, North America, and Australia.

Of 224 responses, 197 (88%) were completed by individuals from different transplant programs, and when analyzing responses by the few individuals from the same center we often found differences in some of the practice areas, with most variability in behaviors such as pre-emptive treatment threshold. The majority of respondents (213; 95%) were involved in adult transplantations. In addition, the majority of clinicians were physicians rather than surgeons (Figures 1B,C). Some respondents managed or conducted several types of SOT. Respondents had been in active clinical SOT practice for a mean of 14.1 years. The median number of transplants reported annually by organ in the respondents’ institutions is shown in Figure 1D: centers volumes indicate that survey participants were mostly practicing in medium to large transplant centers.

When considering European representation, the 128 European hospitals represented in this survey are 40% of the total (316) transplant hospitals active in the 27 European countries included in the survey.

### Cytomegalovirus Diagnosis

The majority of respondents (217/224; 97%) indicated blood CMV-DNA PCR as the tool used to diagnose CMV infection: only 7 (3%) centers used antigenemia. However, the types of assay and units of measurement utilized revealed substantial variability: 124 (57%) used whole-blood PCR and 92 (42%) used plasma PCR. Of note, while 162 respondents (72%) declared that their laboratory used World Health Organization (WHO) standard units to measure DNAemia, only 66 (40%) reported using them for clinical decision making (e.g. threshold for PET initiation) instead of the non-standardized DNA copies/ml. In seven institutions, the WHO standard was not used, and 55 respondents (24%) were unaware as to whether their laboratory used the measurement unit.

### Prevention of Cytomegalovirus Infection/Disease

In total, 193/224 (86%) respondents reported having an established protocol for CMV prevention in SOT recipients, modulated according to organ type in 135 (70%), D/R serostatus in 182 (94%), and use of antithymocyte globulin (ATG) in 114 (59%) of centers. In this context, 31 (13%) of respondents never used PET, and only 10 (5%) never used prophylaxis.

**FIGURE 2:**
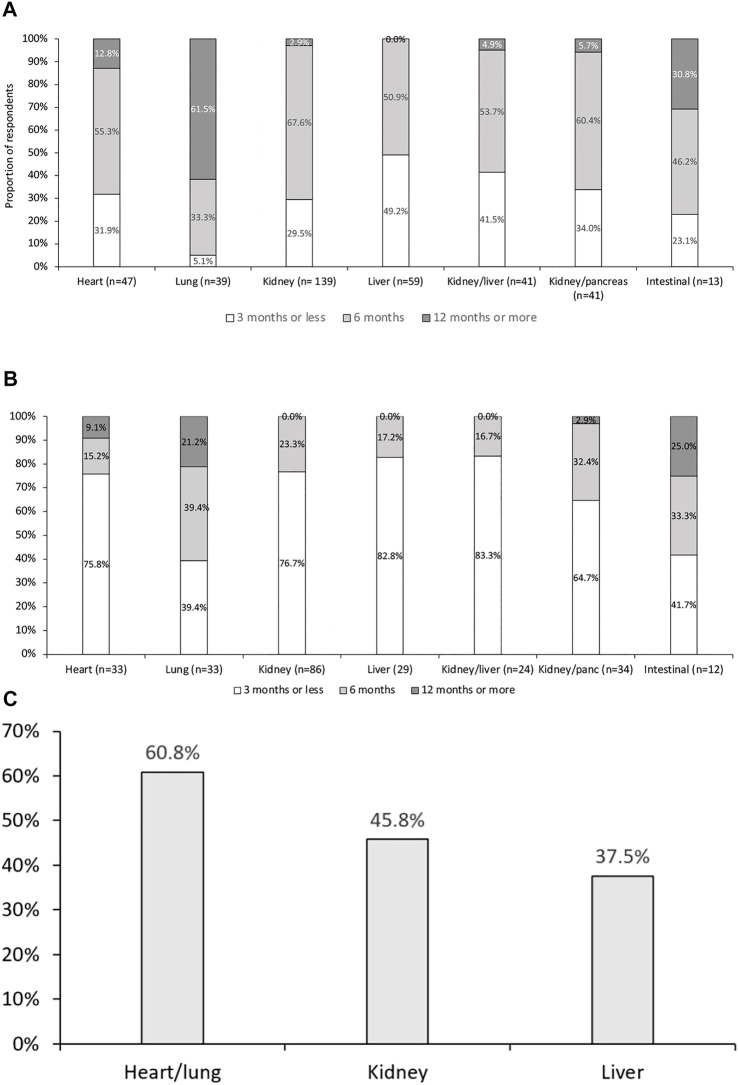
Duration of prophylactic antiviral therapy for cytomegalovirus infection in **(A)** D+/R−, and **(B)** D+/R+. Proportion of respondents using prophylaxis in R+ patients **(C)**. *p* < 0.01 across all groups. After correction for multiple comparisons, duration following lung transplantation was significantly different from all other groups. Antiviral therapy duration following heart transplantation was also significantly different from those following lung and liver transplantation.

Antiviral prophylaxis was administered in D+/R− transplantations by 201 (90%) of respondents (Figure 2A). As expected, prophylaxis was less commonly administered in R+ than in D+/R− transplantations (Figure 2B), and was most commonly administered by respondents performing lung transplantations (Figure 2C).

Prophylaxis use was reported by 99 (44%) of respondents in D+/R+ and 87 (39%) in D−/R+ transplantations. However, 18% and 26% of respondents used neither prophylaxis nor PET in D+/R+ and D−/R+ transplantations, respectively. Conversely, despite D−/R− having the lowest risk of CMV infection, 16% of respondents used prophylaxis in these patients. In this relatively low-risk group, prophylaxis was significantly more commonly utilized in thoracic organ recipients and least utilized in liver transplant recipients (*p* < 0.01) (data not shown).

Prophylaxis duration in R+ recipients was significantly shorter than in D+/R− recipients (Figure 2B). Again, lung transplant recipients had the significantly longest treatment period, with prophylaxis lasting ≥6 months in ∼60% of responses (*p* < 0.01 when compared with abdominal transplantation programs).

While antiviral prophylaxis was evenly distributed across SOT types, duration varied significantly (Figure 2B), with lung transplant specialists using prophylaxis for >12 months in >60% of cases, and liver transplant specialists reporting the shortest duration (all patients <12 months, 49% < 3 months; *p* < 0.01). Of note, 28 (14%) respondents performed CMV surveillance and PET after the end of prophylaxis. Furthermore, there appeared to be no consensus on either the frequency of assessing CMV DNAemia or the duration of PET after prophylaxis.

Valganciclovir was the drug most frequently utilized in prophylaxis regimens. Respondents reported that ∼90% of patients received valganciclovir, while ∼10% of patients might also require intravenous ganciclovir because they were unable to take the oral formulation in the early postoperative period. According to 183 (80%) of respondents, prophylaxis commenced within the first week after transplantation, and 18 (8%) reported the addition of CMVIg for D+/R− patients or those receiving ATG.

**FIGURE 3:**
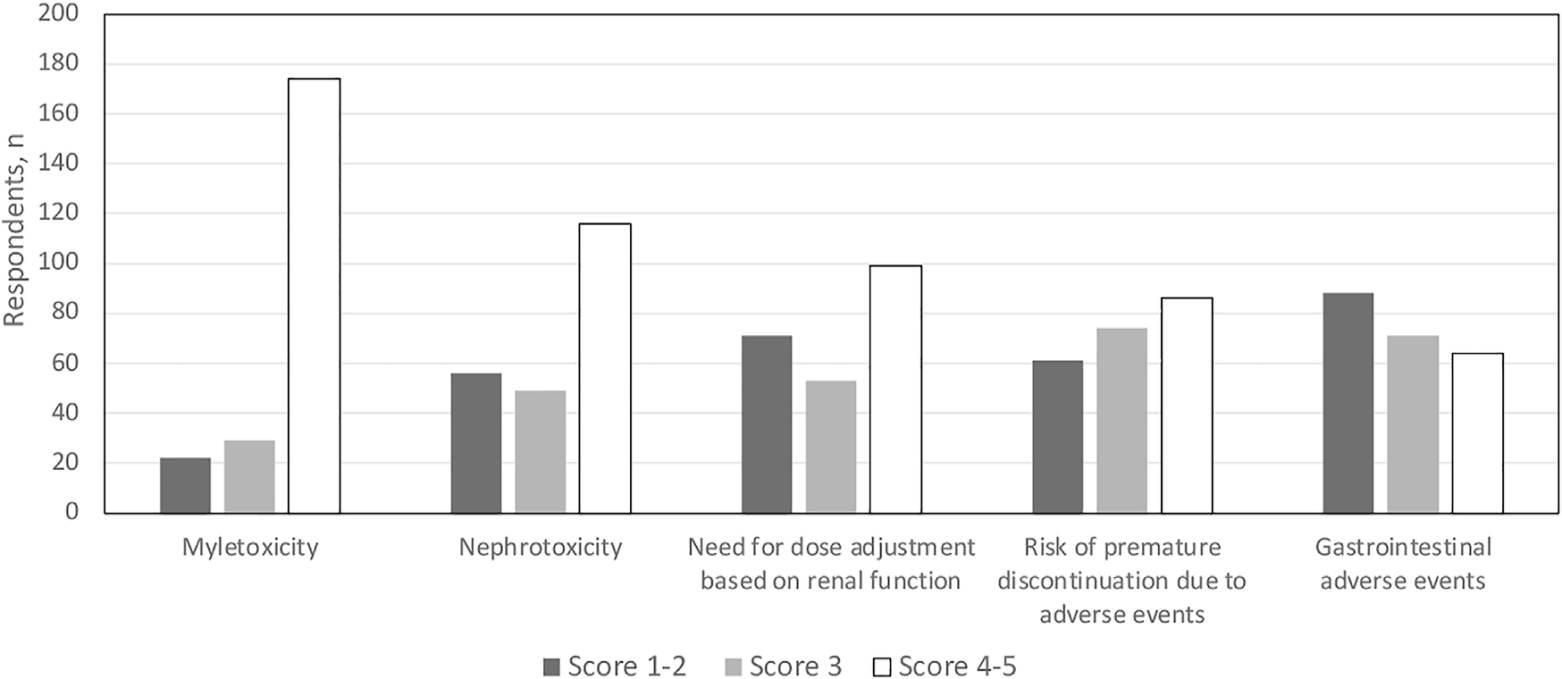
Conditions impacting use of currently approved anti-CMV agents in solid organ transplant recipients. Scores 1–2 do not impact use; score 3 has moderate impact on use; scores 4–5 substantially impact use.

Despite its widespread use, myelotoxicity was considered to have substantial negative impact on valganciclovir administration by 174 (78%) of respondents (leading to drug discontinuation in 10%–20% of SOT recipients, according to 98 [43%] of respondents) (Figure 3). In this regard, treatment of myelotoxicity took a stepwise approach. The most common first step (reported by 100 [44%] respondents) was a reduction in (or withdrawal of) mycophenolic acid derivatives, followed by withdrawal of trimethoprim/sulfamethoxazole. Valganciclovir withdrawal was only considered to be the third step in the approach to myelotoxicity. Of note, 109 (49%) of respondents reported the need for granulocyte colony-stimulating factor in ≥10% of patients because of CMV prophylaxis or PET-related myelotoxicity.

### Pre-Emptive Antiviral Treatment Initiation

**FIGURE 4:**
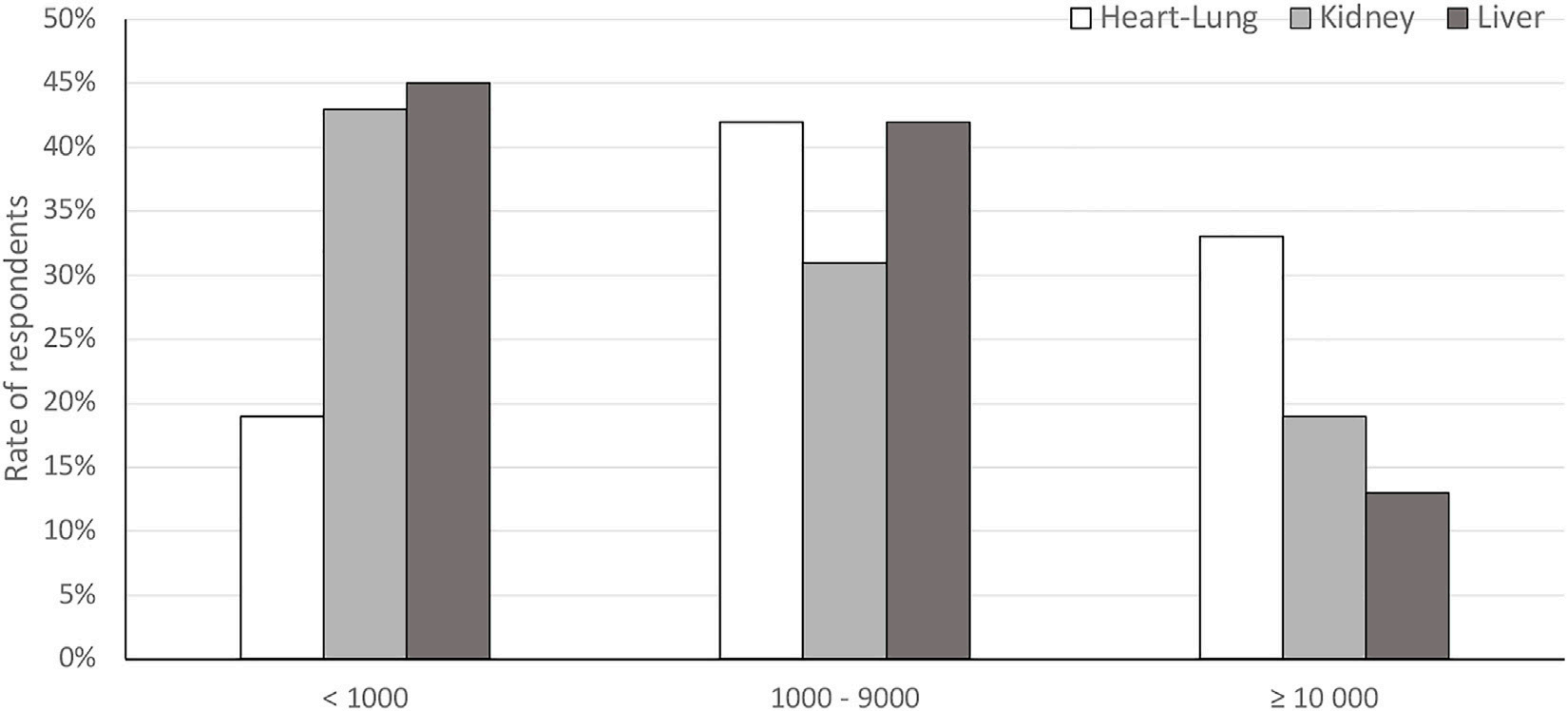
Reported thresholds for pre-emptive antiviral therapy initiation in different organ recipients from respondents using whole-blood polymerase chain reaction assays.

Responses regarding PET management provided a snapshot of the extreme variability and lack of standardization in assay and criteria for treatment initiation. Of 201 respondents indicating DNA thresholds for PET initiation, 58% were based on whole-blood assays; only 66 responses gave thresholds in WHO units. Thresholds were dispersed over a wide range of plasma and whole-blood DNAemia values across all SOT recipients. The median reported plasma DNA threshold value for PET initiation was 1,000 copies/ml (range, 10–10,000 copies/ml), and the median reported whole-blood threshold DNA value was 1,000 copies/ml (range, 5–20,000 copies/ml). Figure 4 shows the distribution of thresholds for whole-blood PCR, which was the most frequently reported assay.

Most respondents (185; 83%) monitored patients after cessation of prophylaxis or PET, with a wide variability of schedules used within 3–6 months after cessation. Values ranged from testing once- or twice-weekly to every 2–3 weeks, depending on time since transplantation and level of infection risk.

PET was widely used, especially in liver recipients: 48% of centers used PET in R+ patients and 12% in D+/R− transplantations. Valganciclovir was the most common first-line PET strategy (reported by 195 [87%] of respondents), followed by intravenous ganciclovir, which was often administered in inpatient settings. In 6% of centers, CMVIg was added to valganciclovir or ganciclovir.

### Treatment of Cytomegalovirus Disease

Treatment of CMV disease was assessed by a question allowing multiple responses. Most respondents (172/224; 77%) indicated intravenous ganciclovir as treatment of choice for CMV disease, but 140 (63%) also administered valganciclovir, supporting an initial uptake of the evidence deriving from the VICTOR trial (23). This survey did not specifically dissect the reasons for choosing intravenous ganciclovir versus valganciclovir, but according to comments added by some respondents it is likely that intravenous ganciclovir was prescribed as the attack strategy, followed by valganciclovir for maintenance; there is no indication if choice was based on disease severity (i.e., end-organ disease versus CMV syndrome), as recommended by current guidelines. Of note, 31 (14%) used CMVIg (in addition to intravenous ganciclovir or valganciclovir) to treat primary CMV infection in cases that involved D+/R− patients, hypogammaglobulinemia (<500 mg/dl), pneumonia, enteritis, or severe leukopenia.

### Treatment Resistance

Molecular diagnostic approaches for detecting CMV resistance were employed by 102/224 (46%) of respondents, while 80 (36%) said that resistance testing was unavailable and 34 (15%) did not know whether testing was available in their institution. Ganciclovir resistance was quite rare, with annual incidence rates of <1% reported by 180 (80%), rates of 1%–5% reported by 39 (17%), and rates of 6%–10% reported by 5 (2%) of respondents.

Infections caused by ganciclovir-resistant CMV strains were treated with high-dose ganciclovir by 109 (49%) of respondents; most of these (157; 70%) used foscarnet, which was usually given following high-dose ganciclovir. A smaller rate of respondents used cidofovir (22%). CMVIg was administered by 69 (31%) of respondents, in combination with antivirals, and 69 (31%) respondents switched patients with infections resultant from ganciclovir-resistant CMV strains to mTOR inhibitors.

Ganciclovir resistance was considered a relevant issue in current CMV management by only 57% of respondents (128 scored ≥5 on a 7-point scale; mean score 4.75). Conversely, when asked about relevant issues for future research, 169 (75%) respondents said that improvement of strategies to manage CMV resistance would be relevant (mean score, 5.29).

### Monitoring Cytomegalovirus-Specific T Cell Response

**FIGURE 5:**
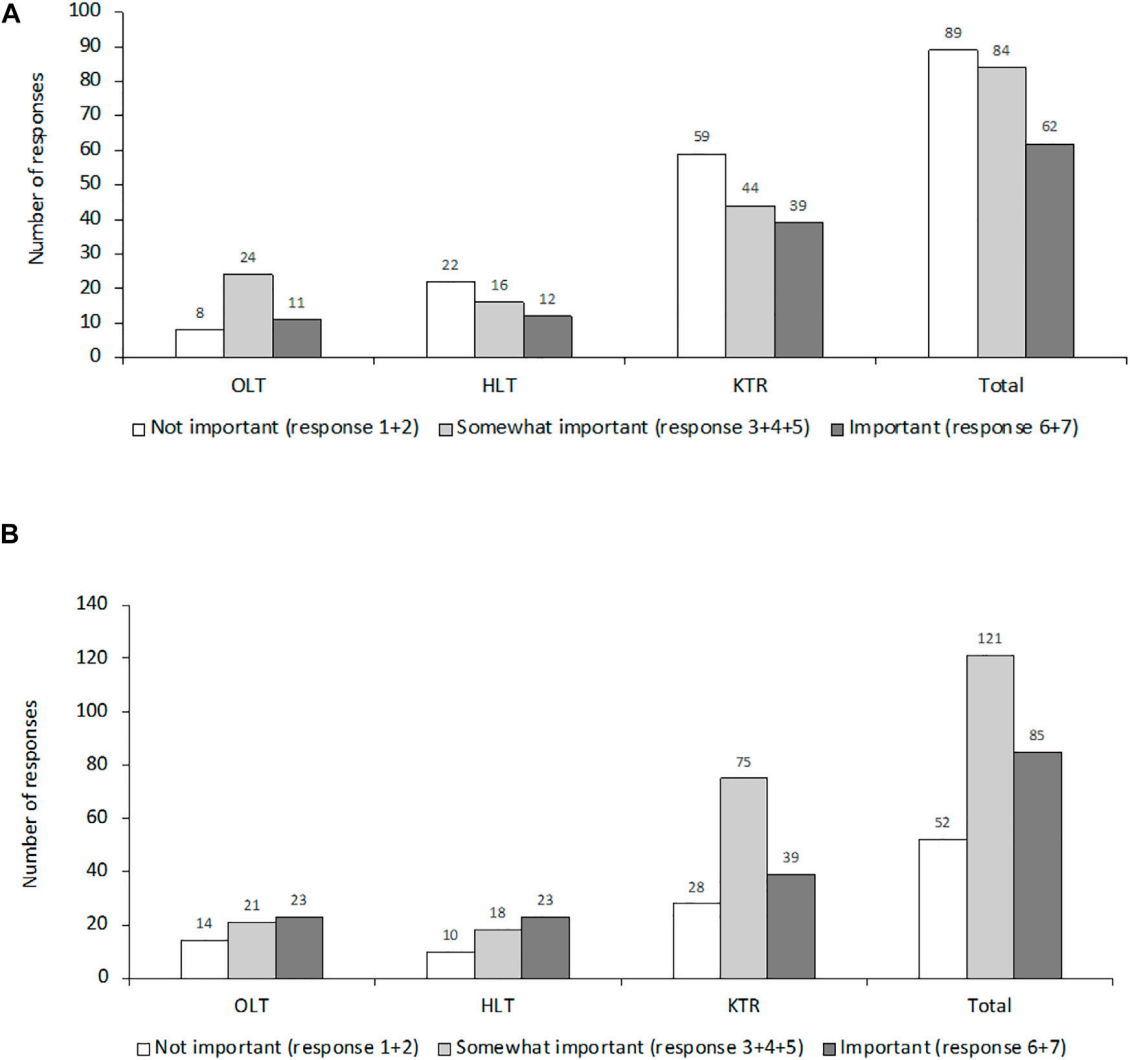
Perception among respondents regarding the importance of CMV-specific T cell response **(A)** at the time of the survey and **(B)** in 1–3 years.

Most respondents (183/224; 82%) said that monitoring of CMV-specific T cell responses was not routinely performed. Centers that offered this facility generally utilized QuantiFERON (26/49; 53%) and/or ELISpot (21/49; 43%). Only 2/49 (4%) utilized other techniques, such as intracellular cytokine staining, MHC multimer, or Viracor. Questions 51 and 52 (Supplementary Table S1) investigated current perceptions of the importance of such analyses and the likelihood that monitoring CMV-specific T cell responses could become standard-of-care in the next 1–3 years. Respondents indicated that immunologic monitoring is not of primary importance but is likely to become more important within the next 1–3 years (Figure 5).

### Key Issues in Cytomegalovirus Management in Solid Organ Transplantation and Future Research

When addressing opinions regarding the major open issues for CMV management, we recorded consensus for “drug toxicities” (5.15), late CMV infection (5.09), and “ease of administration” (5.07). The relevance of “management of CMV resistance” (4.74), “drug cost” (4.57), and “drug interactions” reached lower levels of consensus.

Respondents were asked to indicate what would be relevant for future research and development of CMV in SOT populations (Question 57; Supplementary Table S1). Weighted averages for structured answers ranged from 5.41 to 5.66, indicating consensus that all prespecified topics were considered highly important. The five responses with the highest weighted average scores were “optimizing immunosuppressive protocols” (weighted average 5.66), “long term impact of CMV on graft dysfunction and comorbidities” (5.63), “personalized anti-CMV strategies based on monitoring of CMV-specific T cell response” (5.56), “vaccination” (5.47), and “new drug discovery” (5.41).

## Discussion

This article presents the key findings of an international survey designed to investigate current practices in the management and prevention of CMV infection among members of the ESOT community. Our aims were to assess the distance between current practice and established guidelines, identify educational gaps, explore the unmet needs of currently available treatments, and anticipate the developments in this field.

The findings provide a real-world snapshot, covering a large proportion of the transplant units in Europe, with a glimpse of extra-European practice. As opposed to another recent survey (24), these data capture mostly the opinions of transplant physicians and surgeons in managing CMV infection, with only a small proportion of respondents being infectious disease specialists. The data are nuanced regarding levels of consistency between daily practices, guideline use, and reliance on scientific evidence. While there is an appropriate trend toward a customized approach (related to the difference in CMV risk across the transplanted organs, and donor/recipient serology match), conversely there remains very wide variability in approaches to specific problems including drug resistance, monitoring for infection, and use of laboratory tools to detect CMV-DNA. In addition, the survey revealed some practices clearly not recommendable, based on current evidence.

Prevention and management of CMV infection had a center-specific approach, with some divergence from current guidelines (8,9) (Table 1).

**TABLE 1:** Major discrepancies between guideline recommendations and survey-reported practices.

	Guideline recommendation	Survey-reported practice
CMV-DNA assay	• WHO units for DNAemia are recommended	• Only 40% of respondents use them for decision making
		• 28% don’t know what WHO units are
CMV prophylaxis prescription	• Prophylaxis is NOT recommended in D−/R− patients	• 15% of respondents claim to use prophylaxis in D−/R− patients
CMV prophylaxis duration in D+/R−	• 6 months for KTx	• Most respondents are in line with recommendations, but HTx usually seem to receive longer-term prophylaxis than KTx
	• 3–6 months for HTx and LTx	
	• 6–12 months for LuTx	
Post-prophylaxis DNAemia surveillance	• Suggested only in high-risk patients with high immunosuppression burden	• 12%–16% of respondents perform post-prophylaxis monitoring, but with frequencies between 1 and 3 months
	• Weekly/biweekly frequency is preferred	
CMV resistance	• CMV genotyping is recommended if viral load persists over 6 weeks of adequate GCV administration	• CMV genotyping is unavailable or unknown to 54% of respondents

HTx, heart transplant; KTx, kidney transplant; LTx, liver transplant; LuTx lung transplant.

Regarding diagnostic strategies, CMV-DNA was widely used, with a preference for whole blood as a matrix. Antigenemia was still used by a small minority of centers represented. A previous survey of CMV management in European transplant recipients showed an almost identical proportion of centers using whole blood and plasma (42% vs. 41%, respectively) (24). In our study, a slightly larger proportion of centers utilized whole blood (57% vs. 39% using plasma). In this context, the knowledge and experience in using WHO standard for quantitative DNA PCR was suboptimal (25), identifying an educational gap in awareness and interpretation of laboratory results of viral monitoring.

Current guidelines/recommendations are apparently clear in terms of diagnosis, duration of prophylaxis, and CMV-DNA monitoring, followed by pre-emptive strategies and treatment of CMV disease (8, 9). Nonetheless, few centers strictly followed these guidelines. It is difficult to suggest reasons for this, which in part may be related to budgetary or reimbursement policies in specific countries. Another reason might be the increased usage of mTOR inhibitors and their beneficial effect on CMV replication (26, 27). Finally, there may be some reticence by clinicians to change their usual practice. Nonetheless, these findings clearly outline an educational gap to be filled and provide a foundation on which the limited adherence to management guidelines should be analyzed in greater detail.

Preventive strategies in high-risk (D+/R−) transplantations appeared to rely heavily on valganciclovir prophylaxis. Prophylaxis was used more widely in thoracic organ transplantations than in other SOT procedures, perhaps due to the higher risk of direct and indirect effects of CMV infection in lung recipients, and the fear of indirect CMV effects in heart transplant patients (1, 28).

The observed low rate of prophylaxis in R+ liver transplant recipients is consistent with the perceived lower risk of CMV disease and the reports of lower activity of valganciclovir in these patients (29, 30). Duration of prophylaxis varied across centers, ranging from 3 to 6 months in kidney/liver transplant recipients and from 3 to 12 months in heart/lung recipients. In intermediate- and low-risk groups, the approach to prophylaxis appeared to be heterogeneous in terms of strategy and duration, with most institutes treating prophylactically for up to 6 months. For reasons that are unclear, 25% of respondents reported the absence of a preventive strategy in R+ patients. It was also very surprising that a considerable number of respondents reported using prophylaxis in low-risk patients, an approach not recommended by current guidelines (8, 9).

Although expected, between-center variations in PET cut-off values and sampling schedules were striking. However, most relied on whole-blood (frequently) or plasma (occasionally) CMV-DNA threshold levels in the range of 500–5,000 copies/ml. More than the CMV-DNA value disparity, it is noteworthy that the two matrices were used as synonymous specimens, while it is known that whole blood overall contains 1 log_10_ more CMV-DNA than plasma. Furthermore, ∼40% of centers (those using plasma) were indeed starting PET at a 1 log_10_ higher CMV-DNA level than those using whole blood. These findings reinforce the need for prospective studies to determine the optimum cut-off, sampling schedule, and standardized assay and units of measurement before recommendations for using PET can be decisively made.

Most respondents reported no ganciclovir resistance in their centers, despite extensive use of prophylaxis and the availability of resistance testing. It is unclear whether this reflects the wider real-world situation or represents underestimation specific to this survey. Although a few participants reported using CMVIg in cases of resistance, it is unclear whether this reflects actual documented resistance or merely refractory or recurrent CMV infection. We believe that these uncertainties highlight another educational gap that should be specifically addressed.

Although monitoring the CMV-specific T cell response was not considered crucial, respondents felt it would soon become more important. There is a strong scientific rationale for monitoring the CMV-specific immune response (8, 9), but there are no sufficiently validated procedures to enable this to be done effectively in routine practice. This is therefore another unmet need in CMV management.

The current study had several potential limitations. The survey was advertised to the broad ESOT community, targeting individual healthcare professionals rather than institutions. This approach may have introduced some degree of interest bias by collecting responses from people interested and educated in CMV, while missing data on practices from institutions where CMV infection in SOT is not considered relevant or is not addressed properly. Consequently, we could have missed some education gaps that need to be addressed specifically. Another potential limitation is that some answers were ambiguous. Finally, this survey was conducted in the pre-COVID-19 era, which might have had an impact on the policies within centers, particularly in those using pre-emptive strategies to prevent CMV disease in order to avoid frequent access to the hospital for CMV testing. Nevertheless, this survey highlights a very wide variability in clinical practice, often in discordance with current evidence, thus prompting us to encourage specific education activities to favor a more homogeneous management approach for CMV infection in SOT patients.

Although the burden of CMV in SOT has been alleviated through advances in diagnosis, prevention, and treatment, this viral infection continues to have substantial impact in this patient population. The present survey adds to the body of evidence demonstrating a heterogenous approach to CMV infection management and a divergence from international guidelines. Myelotoxicity is perceived as the major drawback with current agents, underlying the need for novel therapies for prophylaxis, to facilitate a safer and more effective strategy to prevent CMV infection. In this setting, the potential availability of letermovir in SOT may represent a relevant step forward (31–35).

Managing ganciclovir-resistant CMV strains was perceived as a major challenge for most centers. Results of a phase II and III trial with maribavir in refractory/treatment-resistant CMV infection may contribute to improved management of resistance (32, 33).

In conclusion, this study highlights several education gaps and unmet needs in the context of management of CMV infection in SOT. Toxicities of current first-line therapies are a major drawback in clinical practice, while improving the knowledge of WHO standard units for CMV-DNA assays may help to design studies targeted to identify the most appropriate threshold to initiate PET. In this context, further development of assays for CMV-specific immunity could represent a key asset to tailor both PET and prophylaxis approaches (36). Finally, these findings will help to guide the development and promotion of targeted educational activities. The ESOT Working Group will continue this project to try to harmonize and improve the management of CMV infection in this challenging population.

## Data Availability

The raw data supporting the conclusion of this article will be made available by the authors, without undue reservation.
