# Supplementary material for: Cytomegalovirus Management in Solid Organ Transplant Recipients: A Pre-COVID-19 Survey From the Working Group of the European Society for Organ Transplantation
Source: Transpl Int. 2022 Jun 22;35:10332. doi: 10.3389/ti.2022.10332 (PMC9257585; doi:10.3389/ti.2022.10332)
Supplement: Supplementary file 1 [file DataSheet1.pdf]

Cytomegalovirus management in solid organ transplant recipients: A survey from the Working Group of the European Society for Organ Transplantation

Paolo Antonio Grossi[1]*, Nassim Kamar[2], Faouzi Saliba[3], Fausto Baldanti[4,5], Jose M. Aguado[6], Jens Gottlieb[7], Bernhard Banas[8] & Luciano Potena[9]

[1]Department of Medicine and Surgery, University of Insubria, ASST-Sette Laghi, Viale Borri, Italy

[2]Department of Nephrology and Organ Transplantation, CHU Rangueil, Université Paul Sabatier, Toulouse, France

[3]AP-HP Hôpital Paul Brousse, Center Hépato-Biliaire, Université Paris-Saclay, Villejuif, France

[4]Department of Clinical, Surgical, Diagnostic and Pediatric Sciences, University of Pavia, Pavia, Italy

[5]Molecular Virology Unit, Fondazione IRCCS Policlinico San Matteo, Pavia, Italy

[6]Unit of Infectious Diseases, Hospital Universitario "12 de Octobre", School of Medicine, Universidad Complutense, Madrid, Spain

[7]Hannover Medical School, Department of Respiratory Medicine, Hannover, Germany

[8]University Hospital Regensburg, Department of Nephrology, Regensburg, Germany

[9]Heart Failure and Transplant Unit, IRCCS Policlinico di Sant'Orsola, Bologna Academic Hospital, Bologna, Italy

***Correspondence:**
Paolo Grossi

paolo.grossi@uninsubria.it

Supplementary materials
Table S1: List of survey questions.

No.	Question	Possible answers
1	Which of the following best describes your primary medical specialty?	Transplant surgeon<br>Transplant physician, non-surgery<br>Other (please specify)
2	What is your area of expertise? Please check all that apply below.	Heart<br>Lung<br>Liver<br>Kidney<br>Kidney/pancreas<br>Islet<br>Intestinal/composite tissue<br>Other (please specify)
3	What is your area of expertise? Please check all that apply below.	Transplant cardiology<br>Transplant pulmonology<br>Transplant hepatology<br>Transplant gastroenterology<br>Transplant nephrology<br>Transplant infectious disease<br>Transplant virologist<br>Other (please specify)
4	For how many years have you been in active clinical practice (post clinical training) managing SOT recipients?	[Open question]
5	In which country do you practice?	[Open question]
6	In which city do you practice?	[Open question]
7	In which hospital/clinical institution do you practice?	[Open question]
8	In which patient population are you involved?	Adults<br>Paediatric<br>Both
9	Please estimate how many of the following SOT surgical cases your hospital/institution conducts in 1 year (or average per year over the last 3–5 years)? Please also indicate the number of SOT surgical cases and/or SOT recipients for whom you are directly involved in clinical management.	Heart<br>Lung<br>Heart/lung<br>Liver<br>Kidney<br>Liver/kidney<br>Kidney/pancreas<br>Islet<br>Intestine/composite tissue<br>Other* (*Please specify here if you indicated "other" in the table above)<br><br>[Open question in terms of number of SOT cases managed]

Supplementary Materials: Table 1 (list of survey questions)

No.	Question	Possible answers
10	For patients diagnosed with CMV infection (i.e., virus isolation or detection of viral proteins (antigens) or nucleic acid in any body fluid or tissue specimen; including reactivation) OR CMV disease (i.e., CMV end-organ disease or CMV syndrome); OR At risk for CMV infection or reactivation and therefore require CMV prophylaxis or pre-emptive therapy (PET) post-transplant, do you actively:	Prescribe (i.e., write the prescription, or oversee the writing of a prescription by a clinician under your direct supervision)<br>Recommend (in a clinical consultation for SOT recipients) antiviral agents for SOT recipients<br>Oversee the treatment during the follow-up<br>All of the above<br>None of the above
11	In the past three months, for how many SOT recipients did you prescribe or recommend an antiviral agent for EITHER prophylaxis OR PET OR for treatment of CMV infection/disease?	[Open question]
12	Of those patients, how many were prescribed or recommended an antiviral agent?:	For prophylaxis post-transplant CMV?<br>For post-transplant CMV PET?<br>For treatment of CMV syndrome/disease?
13	Which tools are you currently using to diagnose/monitor CMV infections?	Quantitative whole blood DNA PCR<br>Quantitative plasma DNA PCR<br>Antigenemia<br>Other (please specify)
14	Is your laboratory using the WHO standard units for quantitative DNA PCR?	Yes<br>No<br>I don't know
15	Are you performing monitoring after stopping prophylaxis or pre-emptive therapy?	Yes<br>No
16	If yes, please specify frequency and type of patients:	[Open question]
17	What is your threshold of DNA PCR to initiate PET?	[Open question]
18	In which units?	Copies/ml in whole blood<br>Copies/ml in plasma<br>IU/ml in plasma<br>IU/ml in whole blood<br>pp65/100000 WBC
19	Does your medical center have access to molecular diagnostics for detection of CMV resistant strains?	Yes<br>No<br>I don't know
20	If yes, please specify which test:	[Open question]
21	At your primary clinical institution, is there an institutional protocol for CMV prevention for the SOT recipient patient population?	Yes<br>No

Supplementary Materials: Table 1 (list of survey questions)

No.	Question	Possible answers
22	What is the protocol based on?	SOT organ type?<br>Donor/recipient CMV serostatus?<br>ATG induction therapy?<br>Other*<br>*Please specify here if you selected "Other" in the list above
23	According to donor/recipient CMV serostatus, what is your preventive approach for D+R-?	PET<br>Prophylaxis<br>PET after prophylaxis<br>None
24	According to donor/recipient CMV serostatus, what is your preventive approach for D+R+?	PET<br>Prophylaxis<br>PET after prophylaxis<br>None
25	According to donor/recipient CMV serostatus, what is your preventive approach for D-R+?	PET<br>Prophylaxis<br>PET after prophylaxis<br>None
26	According to donor/recipient CMV serostatus, what is your preventive approach for D-R-?	PET<br>Prophylaxis<br>PET after prophylaxis<br>None
27	At your primary clinical institution, which of the following products are listed and/or used as recommended (first-line) on the CMV prophylaxis protocol for the SOT recipient patient population? Please check all that apply.	Valganciclovir<br>IV ganciclovir<br>Valaciclovir<br>CMVIg<br>None - I am not using prophylaxis<br>Other (please specify)
28	If you chose more than one agent, please explain:	[Open question]
29	At your primary clinical institution, which of the following products are listed and/or used as recommended (first-line) on the PET protocol for the SOT recipient patient population? Please check all that apply.	Valganciclovir<br>IV ganciclovir<br>CMVIg<br>None - I am not using PET<br>Other (please specify)
30	If you chose more than one agent, please explain:	[Open question]
31	At your primary clinical institution, which of the following products are listed and/or used as recommended (first-line) on the CMV syndrome/disease protocol for the SOT recipient patient population? Please check all that apply.	Valganciclovir<br>IV ganciclovir<br>CMVIg<br>Other (please specify)
32	If you chose more than one agent, please explain:	[Open question]
33	How often do you see (val)ganciclovir resistance in your patient during a year?	<1%<br>1–5%<br>6–10 %<br>>10%

No.	Question	Possible answers
34	At your primary clinical institution, which of the following products are listed and/or used as recommended on the CMV resistant strains? Please check all that apply.	High dose valganciclovir/IV ganciclovir<br>Foscarnet<br>Cidofovir<br>CMVIg<br>Switch to an mTOR inhibitor<br>Other (please specify)
35	If you chose more than one agent, please explain:	[Open question]
36	Based on your SOT recipient patient population at your institution, how do the following statements reflect your attitudes and/or clinical decisions regarding post-transplant management of CMV infection, reactivation, and/or disease? 1= do not agree at all; 7=completely agree.	I am concerned about CMV reactivation, infection or disease<br>I don't need to be concerned about CMV prophylaxis because there is a low incidence of CMV infection/disease post-transplant at my institution<br>I believe that the incidence of CMV infection/reactivation/disease is increasing<br>I believe that I am able to obtain better clinical outcomes if I reduce the risk of CMV infection/reactivation/disease<br>Of the currently available strategies for CMV management, I believe that prophylaxis is the best clinical approach to reduce the risk of CMV infection/reactivation/disease<br>Of the currently available strategies for CMV management, I believe that pre-emptive therapy is the best clinical approach to reduce the risk of CMV infection/reactivation/disease<br>I believe that following the CMV management guideline recommendations for SOT recipients leads to clinically successful outcomes<br>I am concerned about the short- and long-term effects of CMV infection/reactivation/disease, including mortality and organ function/rejection<br>I believe that the most significant risk factor for CMV infection/ disease is the donor/recipient CMV serostatus.<br>Those who are D+R- are at highest risk for CMV infection/disease<br>CMV prophylaxis has demonstrated an overall long term survival benefit in SOT recipients<br>CMV prophylaxis in SOT recipients reduces overall health costs<br>In my SOT recipient patient population at highest risk for CMV infection/disease, CMV management is clinically challenging

Supplementary Materials: Table 1 (list of survey questions)

No.	Question	Possible answers
		Toxicities associated with currently marketed anti-CMV drugs make it difficult to use them for prophylaxis<br>Toxicities associated with currently marketed anti-CMV drugs make it difficult to use them for PET, and/or treatment of CMV infection/disease<br>Many SOT recipient patients have to prematurely discontinue anti-CMV prophylaxis due to toxicities associated with such anti-CMV agents<br>Many SOT recipient patients have to prematurely discontinue anti-CMV PET/treatment regimen due to toxicities associated with such anti-CMV agents<br>I often have to adjust the dose (i.e. according to renal function) of the anti-CMV medication to avoid toxicities
37	Based on your experiences and perception, which of the following factors are associated with increased risk for developing CMV infection/reactivation/disease?	Donor/recipient CMV serostatus<br>Induction with ATG<br>Induction with IL-2 receptor blockers<br>Cumulative steroid dose<br>Organ type<br>Treatment of acute rejection<br>Graft dysfunction<br>Plasma exchange for antibody mediated rejection<br>Maintenance therapy with mTOR inhibitors
38	For what percentage of SOT recipient patients did you recommend/use the following antiviral agents for CMV prophylaxis in the last 3 months?	Valganciclovir<br>IV Ganciclovir<br>Valaciclovir<br>CMVIg<br>Other*<br>None – I do not prescribe antiviral agents for CMV prophylaxis<br>*Please specify here if you indicated "Other" in the list above
39	When do you start CMV prophylaxis for your SOT recipient patient population?	During surgery (i.e. first dose given intra-operatively)<br>Days 1–3 post-transplant<br>Days 4–7 post-transplant<br>Day 8–10 post-transplant<br>Day 11–14 post-transplant<br>After Day 14 post-transplant<br>I do not use CMV prophylaxis

No.	Question	Possible answers
40	In the table below, please indicate how many months on average you continue CMV prophylaxis medication by patient type? D+R-	Heart<br>Lung<br>Liver<br>Kidney<br>Liver/kidney<br>Kidney/pancreas<br>Islet<br>Intestinal/composite tissue<br>Other*<br>*Please specify here if you indicated "Other" in the list above
41	What percentage of your SOT recipient population would require IV formulation of medication for CMV prophylaxis?	[Open question]
42	Of the total duration of CMV prophylaxis, what percentage of days do you anticipate using:<br><br>(Please make sure that total number adds up to 100%)	Oral formulation<br>IV formulation
43	Please rate how important each attribute below is to you when deciding which antiviral therapy to prescribe / recommend for the prophylaxis of CMV in your SOT recipient patient population. 1=not at all important; 7=extremely important.	Effective in CMV prophylaxis<br>Effective in treating suspected or confirmed CMV infection or disease<br>No dose adjustments necessary for renal impairment (CrCl $\geq$0)<br>No dose adjustments necessary for patients requiring dialysis<br>No dose adjustment for Child-Pugh B hepatic insufficiency<br>No dose adjustment for Child-Pugh C hepatic insufficiency
44	Based on your knowledge of and experience with antiviral agents for the prophylaxis of CMV, please indicate how the agents below perform (i.e., clinical impact based on efficacy and safety). Please provide your answer on a 1 to 7 scale, where 1 = "Performs Extremely Poorly" and 7 = "Performs Extremely Well". You may select "Don't Know" if you have no experience with a particular agent.	Valganciclovir<br>Ganciclovir<br>Valaciclovir<br>CMVIg<br>Other*<br>*Please specify here if you indicated "Other" in the list above
45	When considering the adverse event profile of currently approved/marketed anti-CMV agents, please indicate how significant these drawbacks are when managing your SOT recipients for CMV infection	Myelotoxicity (e.g., leukopenia, neutropenia, anaemia, and/or thrombocytopenia)<br>Nephrotoxicity<br>Gastrointestinal adverse events (e.g., nausea, vomiting, diarrhoea)<br>Requirement for dose adjustment based on renal function<br>Requirement for dose adjustment based on hepatic function

No.	Question	Possible answers
		Risk of premature prophylaxis, PET, and/or treatment discontinuation due to adverse events<br>Other*<br>*Please specify here if you indicated "other" in the list above
46	In what percentage of your patients you have to discontinue CMV prophylaxis due to:	None - I am not using prophylaxis at all<br>Myelotoxicity (e.g., leukopenia, neutropenia, anaemia, and/or thrombocytopenia)<br>Nephrotoxicity<br>Gastrointestinal adverse events (e.g., nausea, vomiting, diarrhoea)<br>Breakthrough CMV infection<br>Other*<br><br>*Please specify if you selected "Other" in the list above
47	In what percentage of your patients you have to discontinue CMV PET due to:	None - I am not using PET at all<br>Myelotoxicity (e.g., leukopenia, neutropenia, anaemia, and/or thrombocytopenia)<br>Nephrotoxicity<br>Gastrointestinal adverse events (e.g., nausea, vomiting, diarrhoea)<br>Not effective in reducing viral load<br>Other*<br><br>*Please specify if you selected "Other" in the list above
48	In case of severe neutropenia (less than 1000/mm3) put in order your actions:	Stop CMV prophylaxis/PET<br>Stop or dose reduce MMF/MPA<br>Stop or dose reduce trimethoprim/sulfamethoxazole<br>Prescribe G-CSF
49	Do you take any other actions? If yes, please explain.	[Open question]
50	During first year post-transplant, including during and after CMV prophylaxis/treatment at your primary clinical institution, what percentage of all your recipients receive 1 or more doses of G-CSF in a given year?	[Open question]
51	At the time of survey completion, how important is monitoring of CMV-specific T cell response to the clinical management of SOT recipients at your primary clinical institution?	1=not at all important; 7=extremely important
52	At the time of survey completion, is monitoring of CMV-specific T cell response (including but not limited to commercially available assays such as	Yes<br>No

No.	Question	Possible answers
	QuantiFERON-CMV and non-commercially available/experimental assays) routinely performed at your primary clinical institution in SOT recipients during or after CMV prophylaxis/treatment?	
53	Which assay do you use at your clinical institution for monitoring CMV specific T cell response?	Quantiferon CMV<br>ELISpot<br>Other (please specify)
54	Over the last 1–2 years, approximately what percentage of all SOT recipients at your primary clinical institution are monitored for CMV-specific T cell immune response?	[Open question]
55	In 1–3 years following the completion of the survey, how likely do you think monitoring of CMV-specific T cell responses will be used as standard of care for post-transplant management of SOT recipients at your primary clinical institution?	1=not at all likely; 7=extremely likely
56	What are the most relevant issues in the CMV management (including CMV prophylaxis/PET/treatment) in the SOT recipient patient population?	Monitoring of CMV-specific T cell response<br>Late CMV infection<br>Management of CMV resistance<br>Drug toxicities<br>Drug cost<br>Drug interactions<br>Ease of administration<br>Insufficient efficacy of current available drugs<br>Others*<br>*Please specify here if you selected "Others" in the list above.<br><br>1=not at all relevant to 7=extremely relevant
57	What do you think will be relevant for future research and development of CMV field in SOT?	Personalised anti-CMV strategies based on monitoring of CMV-specific T cell response<br>Vaccination<br>Management strategies of CMV resistance<br>Optimising immunosuppressive protocols<br>New drug discovery<br>Long term impact of CMV on graft dysfunction and comorbidities<br>Other*<br>* Please specify here if you selected "Others" in the list above.<br><br>1=not at all relevant to 7=extremely relevant
